# The role of polymorphic variants of arginase genes (ARG1, ARG2) involved in beta-2-agonist metabolism in the development and course of asthma

**DOI:** 10.18699/VJ20.631

**Published:** 2020-07

**Authors:** O.N. Savelieva, A.S. Karunas, Yu.Yu. Fedorova, R.R. Murzina, A.N. Savelieva, R.F. Gatiyatullin, E.I. Etkina, E.K. Khusnutdinova

**Affiliations:** Bashkir State University, Ufa, Russia; Bashkir State University, Ufa, Russia Institute of Biochemistry and Genetics – Subdivision of the Ufa Federal Research Centre of the Russian Academy of Sciences, Ufa, Russia; Institute of Biochemistry and Genetics – Subdivision of the Ufa Federal Research Centre of the Russian Academy of Sciences, Ufa, Russia; Bashkir State Medical University of the Ministry of Healthcare of the Russian Federation, Ufa, Russia; Bashkir State University, Ufa, Russia; Bashkir State Medical University of the Ministry of Healthcare of the Russian Federation, Ufa, Russia; Bashkir State Medical University of the Ministry of Healthcare of the Russian Federation, Ufa, Russia; Institute of Biochemistry and Genetics – Subdivision of the Ufa Federal Research Centre of the Russian Academy of Sciences, Ufa, Russia St. Petersburg State University, St. Petersburg, Russia

**Keywords:** asthma, beta-2-agonists, arginase 1 (ARG1), arginase 2 (ARG2), association, predisposition genes, бронхиальная астма, бета-2-агонисты, аргиназа 1 (ARG1), аргиназа 2 (ARG2), ассоциация, гены предрасположенности

## Abstract

Asthma is a common severe disease of the respiratory tract, it leads to a significant impairment in the quality of a patient’s life unless effectively treated. Uncontrolled asthma symptoms are a cause of disease progression and development, they lead to an increase in the patient’s disability. The sensitivity to asthma therapy largely depends on the interaction of genetic and epigenetic factors, which account for about 50–60 % of variability of therapeutic response. Beta-2-agonists are some of the major class of bronchodilators used for asthma management. According to published data, allelic variants of the arginase ARG1 and ARG2 genes are associated with a risk of asthma development, spirometry measures and efficacy of bronchodilator therapy. High arginase activity results in a low level of plasma L-arginine and in a decrease in nitric oxide, and, as a result, in an increase in airway inflammation and remodeling. Arginase genetic polymorphisms (rs2781667 of the ARG1 gene, rs17249437, rs3742879, rs7140310 of the ARG2 gene) were studied in 236 children with asthma and 194 unrelated healthy individuals of Russian, Tatar and Bashkir ethnicity from the Republic of Bashkortostan. Association analysis of the studied polymorphisms with asthma development and course, the sensitivity to therapy in patients was carried out. It was found that the rs2781667*C allele of the ARG1 gene is a marker of an increased risk of asthma in Tatars. In Russians, the association of rs17249437*TT and rs3742879*GG genotypes of the ARG2 gene with a decrease in spirometry measures (FEV1, MEF25) was established. In Russians and Tatars receiving glucocorticoid monotherapy or combination therapy, the association of the rs17249437*T allele and rs17249437*TT genotype of the ARG2 gene with a partially controlled and uncontrolled course of asthma was shown.

## Abstract (ru)

Бронхиальная астма (БА) – широко распространенное тяжелое заболевание дыхательных путей, которое при недостаточно эффективном лечении приводит к значительному ухудшению качества жизни пациентов. Отсутствие контроля над симптомами БА ведет к быстрому прогрессированию, утя- желению заболевания и инвалидизации пациентов. Чувствительность к лекарственной терапии БА во многом зависит от взаимодействия генетических и эпигенетических факторов, которые на 50–60 % опре- деляют вариабельность терапевтического ответа пациентов. Одной из основных групп препаратов, используемых для лечения БА, являются бета-2-агонисты, оказывающие значительное бронходилатирующее действие. По литературным данным, аллельные варианты генов аргиназ ARG1 и ARG2 ассоциированы с риском развития БА, показателями спирографии и эффективностью терапии бронходилататорами. Повы- шенная экспрессия генов аргиназ ведет к снижению биодоступности L-аргинина и уровня оксида азота в организме и, как следствие, к увеличению степени воспаления и ремоделирования дыхательных путей. Выполнено исследование полиморфных вариантов генов аргиназ (rs2781667 гена ARG1 и rs17249437, rs3742879, rs7140310 гена ARG2) у 236 детей, больных БА, а также у 194 неродственных здоровых индиви- дов русской, татарской и башкирской этнической принадлежности, проживающих на территории Респу- блики Башкортостан. Проведен поиск ассоциаций изученных полиморфных вариантов с развитием, тече- нием БА и чувствительностью к терапии у пациентов с БА. Установлено, что аллель rs2781667*С гена ARG1 является маркером повышенного риска развития БА у татар. В группе русских пациентов с БА генотипы rs17249437*TT и rs3742879*GG гена ARG2 ассоциированы со снижением показателей спирографии (ОФВ1, МОС25). У русских и татар, находящихся на монотерапии ингаляционными глюкокортикостероидами или на комбинированной терапии, показана ассоциация аллеля rs17249437*Т и генотипа rs17249437*TT гена ARG2 с частично контролируемым и неконтролируемым течением астмы.

## Introduction

Bronchial asthma (BA) is a heterogeneous chronic respiratory disease that caused by an interaction of genetic and environmental risk factors. The prevalence of asthma in the world is 1–18 %, while a significant proportion of patients have insufficient asthma control (GINA, 2018). Beta-2-adrenergic receptor agonists are one of the main groups of drugs used in asthma treatment. Short-acting beta-2-agonists (SABA) are drugs of choice for the treatment of bronchospasm in acute asthma exacerbations. Long-acting beta-2-agonists (LABA) have anti-inflammatory effects due to reduced vascular permeability, reduced secretion of mediators from mast cells and basophils, and reduced bronchial hyperreactivity in prolonged use by patients (National Program…, 2017).

According to the literature, the contribution of genetic factors to individual response of BA patients to therapy is about 50–60 % (Farzan et al., 2017). More than 20 candidate genes associated with beta-2-agonist sensitivity (ADRB2, CRHR2, ADCY9, ARG1, ARG2, etc.) have been detected (Martinez et al., 1997; Litonjua et al., 2008; Poon et al., 2008; Vonk et al., 2010; Kim et al., 2011; Fedorova et al., 2013; Batozhargalova et al., 2017; Scaparrotta et al., 2019). Genome-wide association studies (GWAS) and the examination of large samples within the framework of international consortia allowed to significantly increase the number of genes and intergenic polymorphisms associated with the effectiveness of bronchodilator therapy (COL22A1, SLC22A15, SLC22A23, OXR1, THRB, NTM, etc.) (www.genome.gwas.org).

For the most studied rs1042713 (с.46A>G, p.Arg16Gly) polymorphism of the beta-2 adrenergic receptor gene ADRB2, involved in metabolism of beta-2-agonists, clinical trial of the third stage was performed (Bateman et al., 2011), and the level 2A annotation of that polymorphism was published on the PharmGB website, proving the practical relevance of asthma pharmacogenetic studies (https://www.pharmgkb.org/gene/PA39/clinicalAnnotation/). Furthermore, an important role of other polymorphisms on the efficacy of beta-2-agonist therapy in asthma patients of different ethnicity has been established. The association of genotypes and haplotypes of the adenylyl cyclase type 9 (ADCY9) gene with improvement of lung function measurements in response to using beta-2-agonists in asthma patients from Korea was identified, besides genetic variants of the thyroid hormone receptor beta (THRB) gene and the corticotropin-releasing hormone receptor 2 (CRHR2) gene were associated with a more significant bronchodilator response in patients from Europe (Kim et al., 2011; Duan et al., 2013; Drake et al., 2014). A number of studies have shown that allelic variants of ARG1 and ARG2 genes are associated with BA development, spirometry measures and the effectiveness of bronchodilator therapy (Li et al., 2006; Salam et al., 2009; Vonk et al., 2010; Duan et al., 2011). The increased expression of arginase genes leads to reduced bioavailability of L-arginine and nitrogen oxide levels in the body, increased production of polyamines and proline, and as a consequence, to increased inflammation and remodeling of the respiratory tract (Li et al., 2006; Cloots et al., 2018; Meurs et al., 2019; Said et al., 2019).

The aim of our research was to analyze the association of arginase 1 ARG1 (rs2781667) and arginase 2 ARG2 (rs17249437, rs3742879, rs7140310) genetic polymorphisms with development and course of asthma in children of different ethnicity.

## Materials and methods

DNA samples of 430 unrelated individuals aged 2–17 years from the Republic of Bashkortostan were used in the present study (Table 1). The group of patients consisted of 236 children with bronchial asthma (70 girls, 166 boys) of different ethnicities (Russians – 84, Tatars – 108, Bashkirs – 44). All examined individuals were patients at the children’s clinic at Bashkir State Medical University of the Ministry of Health of Russia (Ufa, Russia) and the Allergology Department of the Republican Children’s Clinical Hospital (Ufa, Russia). The criteria for inclusion of children in the main observation group included the established diagnosis of “bronchial asthma” in accordance with GINA (Global Initiative for Asthma) criteria and the criteria of Russian program documents on BA diagnosis, treatment, and prevention (National Program…, 2012).

**Table 1.**
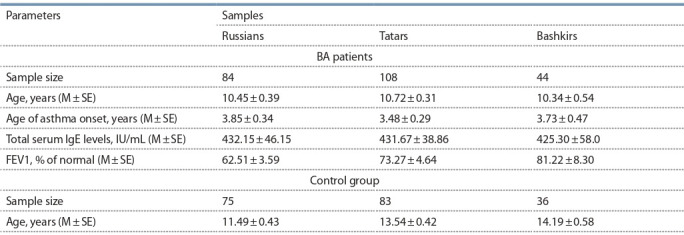
Characteristics of asthma patients and control group Note: M – mean; SE – standard error of mean.

All asthma patients were treated for at least three months with inhaled glucocorticosteroids (ICS) monotherapy or the combination of inhaled glucocorticosteroids and long-acting beta-agonist (ICS–LABA) in a once-daily dose of 100 to 1000 micrograms of fluticasone propionate, depending on the disease severity. The group of asthma patients on ICS monotherapy was included 187 individuals, patient group receiving ICS–LABA combination therapy was composed of 49 individuals. Patient group with controlled asthma on the background therapy by ICS and ICS–LABA was included 172 individuals, group with partially controlled asthma – 50 individuals, group with uncontrolled asthma – 14 individuals.

The evaluation of respiratory function was performed using a computer spirometer (Erich Jaeger, Germany) with flow–volume curve analysis. The following parameters were assessed (in percent of the expected value present in the computer database of the spirometer): vital capacity (VC), forced vital capacity (FVC), forced expiratory volume in 1 sec (FEV1), forced expiratory flow between 25 and 75 % of forced vital capacity (MEF75, MEF50, MEF25, respectively). The normal range and reduction in parameters of spirogram (in percent of the standard value) for children under 18 years were assessed according to Klement and Zilber (1993). Patients with clinical asthma symptoms who were unable to perform spirometry were subjected to multiple measurements of peak expiratory flow rate (PEFR).

Assessment of current level of asthma control on the background of at least 3 months of therapy was carried out on the basis of clinical signs for the last 4 weeks (frequency of daytime symptoms and frequency of night waking up per week, the need for drugs to control attacks in a week, activity restriction due to asthma) by using a validated questionnaire “Asthma Control Test”. A group of apparently healthy children without bronchopulmonary, allergic, and autoimmune diseases and any familial history of allergic diseases consisting of 194 individuals (119 girls, 75 boys) of the corresponding ethnicity (75 Russians, 83 Tatars, 36 Bashkirs) served as a control. Children in the control group had low levels of immunoglobulin E (IgE) and no deviations from normal respiratory function according to spirometry or picfluometry data. An informed consent to participate in the study was obtained from all the children over 15 years and parents of children under 15 years participating in the study. The study protocol was approved by the local Bioethical Committees at the Bashkir State Medical University (Protocol no. 28 dated October 29, 2012) and the Institute of Biochemistry and Genetics of the Ufa Federal Research Centre of the Russian Academy of Sciences (Protocol no. 4 dated November 15, 2012).

Genomic DNA was isolated from peripheral blood lymphocytes by phenol-chloroform extraction (Mathew, 1984). Analysis of the rs2781667 (c.57+665C > T) polymorphism of the arginase 1 ARG1 gene and rs17249437 (c.185-8016T > C), rs3742879 (c.859+101A > G), rs7140310 (c.363-1623T > G) polymorphisms of the arginase 2 ARG2 gene was carried out using DNA amplification by the polymerase chain reaction (PCR) with fluorescent detection (FLASH/RTAS) (TestGen, Moscow) according to the manufacturer’s protocol using the CFX96 real-time PCR detection system (Bio-Rad, USA).

Selection of single-nucleotide polymorphisms (SNP) in studied genes was based on literature data, information from databases about variation allele frequencies (over 5 %), their possible regulatory influence on gene expression and functional significance (Li et al., 2006; Salam et al., 2009; Vonk et al., 2010; Duan et al., 2011).

The χ2 criterion was used to verify the correspondence of the observed distribution of genotype frequencies to the expected one according to the Hardy–Weinberg equilibrium. A pairwise comparison of allele and genotype frequencies between the patients and controls was based on the χ2 criterion for 2 × 2 contingency tables with Yates correction. In the case of significant differences in the studied samples, the odds ratio (OR) and the boundaries of 95 % confidence interval (95 % CI) were estimated. Statistical analysis of quantitative data was performed using parametric and nonparametric tests depending on the scales and the distribution of variables via SPSS v.23 (SPSS Inc.). The distribution of quantitative data was assessed according to the Kolmogorov–Smirnov criterion. The equality of general variances was assessed using Levene’s test. Nonparametric tests (Mann–Whitney t criterion and Kruskal–Wallis H criterion) were used in similar comparisons in the case of abnormal distribution or failed equality of variances. The linkage disequilibrium between polymorphisms was estimated by applying the D′ coefficient, proposed by Lewontin, and Pearson correlation coefficient r2. The EMalgorithm realized in program Haploview version 4.2 was used for definition of haplotype frequencies and for testing of differences in haplotype frequencies distributions (https://www.broadinstitute.org/haploview/haploview).

## Results

Allele and genotype frequencies of four polymorphisms of arginase ARG1 (rs2781667) and ARG2 (rs17249437, rs3742879, rs7140310) genes were analyzed in asthma patients and healthy individuals from the Republic of Bashkortostan (Table 2). The distribution of genotype frequencies in all polymorphisms corresponded to the Hardy–Weinberg equilibrium ( p > 0.05). The association analysis of the studied polymorphisms with asthma development, with clinical and functional parameters of BA (degree of asthma control, age of asthma onset, level of total IgE, spirometry parameters) was carried out.

**Table 2.**
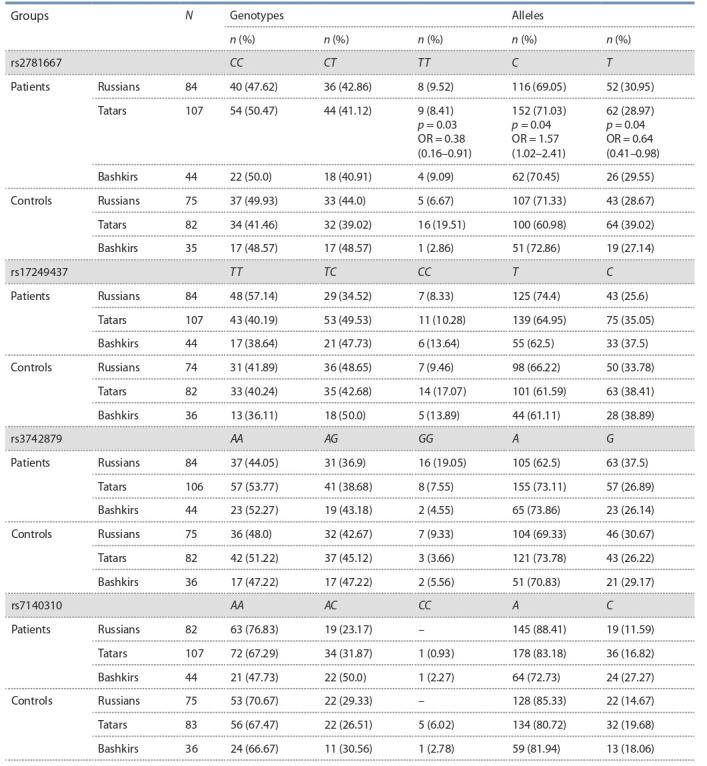
Distribution of allele and genotype frequencies of ARG1 rs2781667, ARG2 rs17249437, rs3742879, rs7140310 gene polymorphisms in asthma patients and control group Note: N is the number of individuals; n is the sample size; alleles and genotype frequencies are shown in brackets; p is the P-value and is shown in the case of statistical significance ( p ≤ 0.05); OR is the odds ratio and 95 % confidence interval (in brackets).

The arginase 1 (ARG1) gene is located on the chromosome 6 (6q23.2) and contains 8 exons (Vonk et al., 2010). The rs2781667*T allele frequency in control groups were as follows: 28.67 % in Russians, 39.02 % in Tatars, 27.14 % in Bashkirs. An association of the rs2781667*C allele with asthma in Tatars ( p = 0.04; OR = 1.57; CI 95% 1.02–2.41) was established. The rs2781667*TT genotype and the rs2781667*T allele are markers of reduced risk for asthma development in Tatars ( p = 0.03; OR = 0.38; CI 95% 0.16–0.91 and p = 0.04; OR = 0.64; CI 95% 0.41–0.98, respectively).

The contribution of allelic variants of studied candidate genes to the variability of quantitative traits (IgE level, age of disease onset) was determined by Kruskal–Wallis test (in case of three groups) or Mann–Whitney test (in case of two groups). The analysis of variations in IgE levels among asthma patients of Russian ethnicity with different genotypes of the rs2781667 polymorphism of the ARG1 gene demonstrated higher IgE values in patients bearing the rs2781667*CC genotype compared to patients with rs2781667*CT and rs2781667*TT genotypes. As a result of pairwise comparison of groups, a statistically significant increase of IgE level was observed in patients bearing the rs2781667*CC genotype compared with individuals with the rs2781667*CT genotype ( p = 0.003).

**Table 3.**
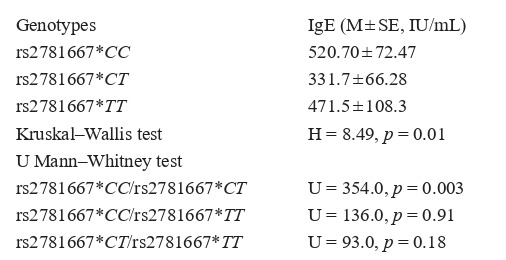
Table 3.

The ARG2 gene is located at the chromosome region 14q24.1 and contains 8 exons (Vonk et al., 2010). An analysis of allele and genotype frequency distributions of the ARG2 polymorphism rs17249437 showed that the rs17249437*C allele was less prevalent in control groups of Russians, Tatars and Bashkirs (33.78, 38.41 and 38.89 % respectively) (see Table 2). No statistically significant associations of the ARG2 polymorphism rs17249437 with asthma were found ( p > 0.05). Differences in genotype distribution of rs17249437 polymorphic variant while dividing patients with account for deviations from normal spirometry values in comparison to the controls were revealed. The frequency of the homozygous rs17249437*TT genotype (67.74 and 67.74 %) in Russian asthma patients with significant decreases FEV1 and MEF25 was significantly higher than in the control group of individuals (41.89 %; p = 0.02; OR = 2.91; CI 95% 1.2–7.05 and p = 0.02; OR = 2.91; CI 95% 1.2–7.05, respectively). The frequency of the heterozygous rs17249437*TC genotype in Russians with significantly reduced FEV1 and MEF25 was lower (19.35 and 22.58 %) than in the controls (48.65 %; p = 0.005; OR = 0.25; CI 95% 0.09–0.69 and p = 0.01; OR = 0.31; CI 95% 0.12–0.8, respectively).

The increased frequency of the rs17249437*T allele (87.93 %) and the rs17249437*TT genotype (82.76 %) was detected in Russian patients with partially controlled and uncontrolled asthma, who required treatment with beta-2- agonists more often than three times a week, compared to patients with controlled asthma: 67.27 % for the rs17249437*T allele ( p = 0.004; OR = 3.54; CI 95% 1.46–8.59) and 43.64 % for the rs17249437*TT genotype ( p = 0.0006; OR = 6.20; CI 95% 2.06–18.64). A similar association has been found in asthma patients of Tatar ethnicity. The rs17249437*T allele (77.59 %) and the rs17249437*TT genotype (58.62 %) were detected more frequently in patients with partially controlled and uncontrolled asthma, in comparison with children with controlled asthma: 60.26 % for rs17249437*T allele ( p = 0.02; OR = 2.28; CI 95% 1.14–4.58) and 33.33 % for rs17249437*TT genotype ( p = 0.02; OR = 2.83; CI 95% 1.18–6.80).

The study results of the ARG2 polymorphism rs3742879 in children with asthma and individuals of the control group from the Republic of Bashkortostan are presented in Table 2. The rs3742879*G allele is less common in all ethnic groups, it was revealed with a frequency of 30.67 % in Russians, 26.22 % in Tatars and 29.17 % in Bashkirs control groups. The association analysis of the ARG2 polymorphism rs3742879 with asthma in individuals of different ethnic origin did not reveal statistically significant differences between groups of patients and controls ( p > 0.05). A comparative analysis of allele and genotype frequencies of the rs3742879 polymorphism in asthma patients with different spirometry measures revealed that the rs3742879*GG genotype was significantly more frequently (25.81 %) in Russians with significant decrease of FEV1 compared to the control group (9.33 %, p = 0.03; OR = 3.38; CI 95% 1.1–10.35).

The study of the ARG2 polymorphism rs7140310 did not identify statistically significant differences in the allele and genotype frequency distributions between asthma patients and controls of different ethnicity ( p > 0.05). The minor allele frequencies (MAF) in control groups were as follows: 14.67 % in Russians, 19.28 % in Tatars, 18.06 % in Bashkirs.

The analysis of rs17249437, rs3742879, rs7140310 polymorphisms of the ARG2 gene in samples of different ethnicity showed significant linkage disequilibrium between rs17249437 and rs3742879 polymorphisms (in Russians Dʹ = 0.76, in Tatars Dʹ = 0.85, in Bashkirs Dʹ = 0.9) in all studied groups. Haplotype analysis of ARG2 gene polymorphisms did not find statistically significant differences in haplotype frequencies between asthma patients and the control group ( p > 0.05).

## Discussion

Insufficient control of inflammation in respiratory tract at asthma leads to the disease progression, contributes to increasing the number of severe forms, deaths, and disabilities of patients. Currently, highly effective drugs have been developed and available, many significant mechanisms of asthma pathogenesis have been discovered, but the problem of insufficient asthma control remains one of the most important public health problems. Many studies provide evidence of interethnic differences in genetic markers for the asthma development and for drug susceptibility in different individuals (Martinez et al., 1997; Litonjua et al., 2008; Poon et al., 2008; Vonk et al., 2010; Kim et al., 2011; Batozhargalova et al., 2017; Scaparrotta et al., 2019). An association of the rs1042713*A allele of the ADRB2 gene with moderate reduction of FEV1 in asthma patients from the Republic of Bashkortostan was found in our earlier study (Fedorova et al., 2013). In this paper, we analyzed associations of arginase ARG1 (rs2781667) and ARG2 (rs17249437, rs3742879, rs7140310) gene polymorphisms with asthma development and course and with sensitivity to therapy in patients.

Arginase is an enzyme that catalyzes the hydrolysis of L-arginine to produce ornithine and urea (Dimitriades et al., 2014). Two isoforms of arginase (types I and II) are encoded by ARG1 and ARG2 genes (Vonk et al., 2010). Allergic inflammation causes a decrease in the total amount of NO and an increase in the production of pro-contact and pro-inflammatory peroxynitrite (ONOO–), in particular iNOS, which leads to obstruction, inflammation and increase in airway hyperreactivity. Moreover, allergic asthma under the influence of Th2-cytokines (IL-4, IL-13) and TGF-β increases the arginase expression, which raises the L-ornithine, polyamines and L-proline production, which participates in airway remodeling, by inducing cell proliferation, and enhanced collagen production and fibrosis (see the Figure) (Meurs et al., 2019).

This research revealed that the rs2781667*C allele of the ARG1 gene is associated with asthma in Tatars. Comparative analysis of qualitative traits detected higher IgE values in Russian patients with the rs2781667*CС genotype compared to children bearing the rs2781667*CT genotype. According to literature, the association of ARG1 gene polymorphisms with the effectiveness of asthma therapy was first established by Litonjua A. et al. in asthma children of European origin treated with beta-2-agonists. The authors found a significant association of the rs2781659*G allele of the ARG1 gene with a more pronounced bronchodilatation response, and the ARG1 gene was proposed as a possible risk marker to determine the effectiveness of asthma therapy (Litonjua et al., 2008). Vonk et al. (2010) found a significant decrease of bronchodilatation response to beta-2-agonists in asthma patients from Netherlands with the rs2781667*TT genotype of the ARG1 gene. At the same time, inhaled glucocorticosteroids slowed down the annual FEV1 decline, which was significantly less effective in homozygote carriers of the C allele at rs2781667 polymorphism in the ARG1 gene (Vonk et al., 2010). An association of the rs2781666*T allele and CT haplotype (rs60389358, rs2781666) of the ARG1 gene with asthma and a considerably higher level of serum arginase was established in asthma patients from India (Donthi et al., 2018). On the contrary, some studies did not detect a significant association of ARG1 gene polymorphisms with the efficacy of asthma therapies (Almomani et al., 2019; Scaparrotta et al., 2019). Together, this results and literature data confirm that ARG1 gene allelic variants can contribute to asthma pathogenesis and the effectiveness of disease therapy.

In the present study associations of rs17249437*TT and rs3742879*GG genotypes of the ARG2 gene with reduced spirometry values (FEV1, MEF25), of the rs17249437*T allele and the rs17249437*TT genotype of the ARG2 gene with partially controlled and uncontrolled asthma were found in patients of Russian and Tatar ethnicity. Contrary, Vonk et al. revealed associations of the rs17249437*T allele, rs3742879*G allele and rs7140310*TT genotype with higher FEV1 in adult asthma patients from Netherlands. It was found association of the rs3742879*AA genotype with increased bronchial hyperresponsiveness (Vonk et al., 2010). In addition, our findings are partially inconsistent with studies Batozhargalova et al., that did not find an association of a combination of NOS2A*(CCTTTT)nS/L and rs3742879*AA genotypes of the ARG2 gene with asthma in girls from Russia (Batozhargalova et al., 2017). Salam et al. in the study of ARG2 gene polymorphisms found an association of the rs3742879*G allele in the haplotype structure (TAGTCATGC, rs12885261, rs7144243, rs3759757, rs4902501, rs7156352, rs4902503, rs7140310, rs742869, rs3742879, rs10483801) with asthma in patients of European origin (Salam et al., 2009). Allelic variants of the ARG2 gene specifically in the haplotype structure might represent an important risk factor of asthma development and progression. In our study of ARG2 gene polymorphisms in samples of different ethnicity, we found a significant linkage disequilibrium between rs17249437 and rs3742879 polymorphisms in all studied groups. Haplotype analysis did not reveal any significant associations of studied ARG2 gene polymorphisms, which is probably due to the small number of compared groups.

**Figure 1.**
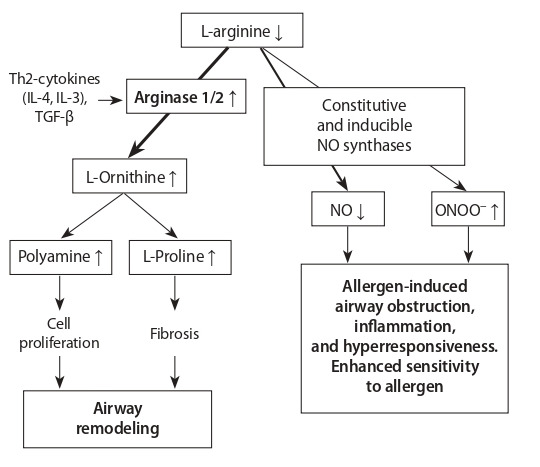
Pathways of arginine metabolism and their relationship to allergen-induced airway obstruction, airway inflammation, airway hyperresponsiveness, airway remodeling, and enhanced allergen sensitivity (Meurs et al., 2019).

## Conclusion

Thus, an association study of polymorphic variants of ARG1 and ARG2 genes involved in beta-2-agonists metabolism was carried out in asthma patients and corresponding control groups from the Republic of Bashkortostan. It was found that the rs2781667*C allele of the ARG1 gene is a marker of increased risk of asthma development in Tatars. Higher values of total IgE were revealed in Russian patients with the rs2781667*CC genotype. It was found that rs17249437*TT and rs3742879*GG genotypes of the ARG2 gene were associated with a decline in lung function (FEV1, MEF25) in Russian patients. In asthma patients of Russian and Tatar ethnicity association of the rs17249437*T allele and the rs17249437*TT genotype with partially controlled and uncontrolled asthma was established. The results obtained in the present study made it possible to thoroughly understand the molecular basis of BA pathogenesis and to identify genetic markers of efficacy of bronchodilator therapy in BA patients.

## Conflict of interest

The authors declare no conflict of interest.
